# Meta-analysis of long-term joint structural deterioration in minimally treated patients with rheumatoid arthritis

**DOI:** 10.1186/s12891-016-1195-4

**Published:** 2016-08-18

**Authors:** Jeroen P. Jansen, Maria-Cecilia Vieira, John D. Bradley, Joseph C. Cappelleri, Samuel H. Zwillich, Gene V. Wallenstein

**Affiliations:** 1 Tufts University School of Medicine, Boston, MA USA; 2 Formerly at Mapi, Boston, MA USA; 3 Pfizer Medicines Development Group, Groton, CT USA; 4 1714 Stockton Street, 3rd floor, San Francisco, CA 94133 USA

**Keywords:** Joint erosions, Longitudinal studies, Meta-analysis, Radiography, Rheumatoid arthritis

## Abstract

**Background:**

Rheumatoid arthritis (RA) is a chronic autoimmune disease characterized by inflammation and joint structural deterioration. Driven by recent expectations that patients in clinical trials randomized to placebo should be ‘rescued’ with active therapy within 6 months of starting treatment, the relative benefit of arresting joint damage with biologic agents beyond this period is unclear. With longer-term evidence of the rate of joint deterioration with minimal treatment, the efficacy of biologic agents and novel treatments might be projected beyond the placebo-controlled phase observed in clinical trials. The aim of this study was to estimate radiographic structural deterioration over time in patients with moderate-to-severe RA minimally treated with DMARDs.

**Methods:**

A literature review identified evidence of joint structural deterioration in patients with (DMARD-IR population) and without (non-DMARD-IR population) a history of inadequate response to DMARDs. Patients were minimally treated with one non-biologic DMARD or palliative care (non-DMARD-IR population only). Outcomes of interest were the (modified) Total Sharp Score (TSS) and subscales (Erosion Subscore [ES] and Joint Space Narrowing [JSN] Subscore), and Larsen score. Pooled joint-deterioration curves over time were obtained with meta-analysis models.

**Results:**

Mean change from baseline in TSS increased in the DMARD-IR population from 1.14 (95 % credible interval [CrI] 0.66, 1.67) to 9.84 (5.68, 14.46) at Weeks 12 and 104, respectively, and a non-linear increase of 1.56 (0.79, 2.34) and 5.13 (−1.35, 11.67) in the non-DMARD-IR population. At the same time points, mean changes (95 % CrI) were 0.51 (0.27, 0.83) and 4.43 (2.38, 7.21) for ES and 0.36 (0.09, 0.67) and 3.14 (0.80, 5.78) for JSN in the DMARD-IR population, whereas corresponding changes in the non-DMARD-IR population were 0.69 (0.31, 1.12) and 2.93 (0.92, 5.02), and 0.29 (0.17, 0.44) and 2.55 (1.45, 3.80), respectively. Larsen scores were only available for the non-DMARD-IR population, with mean changes (95 % CrI) of 0.08 (0.04, 0.11) and 0.65 (0.36, 0.96) at Weeks 12 and 104, respectively.

**Conclusion:**

Minimal treatment of RA with one non-biologic DMARD results in deterioration of joint structure in patients with or without a history of inadequate response to non-biologic DMARDs.

**Electronic supplementary material:**

The online version of this article (doi:10.1186/s12891-016-1195-4) contains supplementary material, which is available to authorized users.

## Background

Rheumatoid arthritis (RA) is a common chronic inflammatory joint disorder. Without treatment most patients with RA become severely disabled. The goals of RA treatment are to reduce disease activity, reduce or inhibit the rate of joint damage and, if possible, achieve remission. Current pharmacologic therapies include traditional disease-modifying anti-rheumatic drugs (DMARDs) and biologic agents [1–3].

Biologic agents have been shown to inhibit radiographic joint destruction in patients with an inadequate response to non-biologic DMARDs. Driven by recent expectations that patients in clinical trials randomized to placebo should be ‘rescued’ with active therapy within 6 months of starting treatment, the relative benefit of arresting joint damage with biologic agents beyond this period is unclear. With longer-term evidence of the rate of joint deterioration with placebo or minimal treatment, the efficacy of biologic agents and novel treatments might be projected beyond the placebo-controlled phase observed in clinical trials.

The objective of the current study was to estimate radiographic joint destruction over time with minimal treatment among the following populations of biologic DMARD-naïve RA patients: (1) moderate-to-severe RA patients with a history of inadequate response to non-biologic DMARDs who were treated with one (other) non-biologic DMARD; and (2) moderate-to-severe RA patients without a history of inadequate response to a DMARD, who received palliative care (non-steroidal anti-inflammatory drugs [NSAIDs], analgesics, low-dose glucocorticoids) or were being minimally treated with one non-biologic DMARD. The first population was termed the “DMARD-IR population” and the second population the “non-DMARD-IR population”. The evidence for this analysis was obtained by means of a systematic literature review.

## Methods

### Study identification and selection

A systematic literature search was performed to identify studies that provided information concerning joint structural deterioration among minimally treated RA patients. MEDLINE® and EMBASE® databases were searched simultaneously for articles published in English, French, or German, from 1970 to October 2009, with a predefined search strategy. Search terms included a combination of free text and thesaurus terms related to RA, NSAIDs, glucocorticoids, non-biologic DMARDs, clinical trials, and observational studies. (See Additional file 1 for details of the search strategy.) The relevance of each citation identified from the databases was based on the title and abstract according to the predefined selection criteria outlined below:

#### Populations of interest

DMARD-IR, i.e., adult RA patients naïve to biologic DMARDs with a history of inadequate response to one or more non-biologic DMARDs; and non-DMARD-IR, i.e., adult RA patients naïve to biologic DMARDs without a history of inadequate response to a non-biologic DMARD. The non-DMARD-IR population could include both non-biologic DMARD-naïve (completely DMARD-naïve) and non-naïve (non-biologic DMARD-experienced) patients.

#### Interventions

NSAIDs, glucocorticoids, and single non-biologic DMARDs, including methotrexate (MTX), azathioprine (AZA), sulfasalazine (SSZ), leflunomide (LEF), ciclosporin A (CSA), hydroxychloroquine (HCQ), minocycline, D-penicillamine, and gold salts.

#### Outcomes

Radiographic measures of joint deterioration: Larsen score (0–200 points range) [4, 5] and Total Sharp Score (TSS) (0–448), plus two TSS subscores (Erosion Subscore [ES] (0–280) and Joint Space Narrowing [JSN] (0–168) Subscore) [6–10].

#### Study design

Randomized controlled trials (RCTs), and prospective and retrospective observational cohort studies. Only study arms concerning the interventions of interest were included.

Publications were obtained, if available, for any abstracts that potentially met the selection criteria. Based on these full-text reports, two reviewers evaluated whether each study met the selection criteria and any disagreements were resolved in a consensus meeting.

### Data extraction

For each of the selected studies that reported sufficient follow-up data, details were extracted from the relevant study arms on study design, population characteristics, interventions, and the outcomes of interest, i.e., the (modified) TSS and its two subscores (ES and JSN). Data were extracted into a study database according to the Preferred Reporting Items for Systematic Reviews and Meta-analyses (PRISMA) 2009 [11]. Mean change from baseline (CFB) in the outcomes of interest was extracted from tables, text, or graphs. If not reported, CFBs were calculated as the difference between reported follow-up and baseline values. Corresponding standard errors were extracted directly or calculated indirectly based on the following data (if available): reported standard deviation (SD) with sample size, 95 % confidence interval, or *p*-values (in this order of preference).

Larsen scores were not consistently evaluated or reported. Different numbers and sets of joints were evaluated in the various studies, including hands and feet, or hands, feet, and wrists, and many studies did not report which or how many joints were evaluated. Moreover, some studies reported the total scores and some reported an average of scores per joint. Consequently, the analyses were based on standardized mean CFB in Larsen score, calculated as the reported CFB divided by the corresponding SD of this change.

### Meta-analysis of joint structural deterioration over time

Mean CFB in TSS, ES, JSN, and the standardized Larsen scores obtained from the selected studies were combined with Bayesian random-effects meta-analysis models to estimate joint deterioration over time for the DMARD-IR and non-DMARD-IR populations [12]. Any study that did not explicitly state whether or not patients had previously shown an inadequate response to DMARDs was assigned to the non-DMARD-IR population. Depending on the availability of data by endpoint and population, two sets of analyses were performed. In the first series of analyses, all non-biologic-DMARDs were considered as one group and the development of the outcomes of interest was estimated. Studies evaluating only NSAIDs were not combined with studies evaluating DMARDs. In the second series of analyses, the development of the outcome over time was compared among individual DMARD (e.g., MTX, LEF, and AZA) using only data from comparative studies. All analyses provided curves reflecting the pooled mean CFB in TSS, ES, JSN, and the standardized Larsen score over time, along with their respective 95 % credible intervals (95 % CrIs).

Within the Bayesian framework, analyses consisted of data, likelihood, parameters, and a model. Bayesian methods involve a formal combination of a prior probability distribution (that reflects a prior belief of the possible values of the parameters of interest) with a likelihood distribution based on the observed data, to obtain a posterior probability distribution of the parameters of interest [13]. A normal likelihood distribution was assumed.

We opted for statistical models that assume that outcomes develop over time in a linear fashion, as well as models that anticipate that outcomes can develop in a non-linear fashion over time [14–16]. The advantage of the used meta-analysis models is that all available data points of each study included in the analysis are captured, even if time points are not the same across studies, and (non-) linear trends of the development of outcomes over time are estimated [12]. Details of the meta-analysis models are provided in Additional file 2. Model 1 and 2 were used to estimate the development over time where all non-biologic DMARDs were grouped. Model 3 and 4 were used for the comparative analysis of different DMARDS. The deviance information criterion (DIC) provides a measure of model fit that penalizes model complexity and was used to compare the different models [17, 18]. The model with the lowest DIC and, therefore the model with the “best fit”, was considered the most appropriate.

To avoid prior beliefs influencing the results of the model, non-informative prior distributions were used. Prior distributions of all model parameters were normal distributions with a mean of 0 and a variance of 10^4^, except for heterogeneity, which was a uniform distribution with a range of 0–10. With such a “flat” prior, it is assumed that, in advance of the actual data, any parameter value is “equally” likely. As a consequence, posterior results are not influenced by the prior distribution but are driven by the data. The result of the Bayesian analysis is a (joint) posterior distribution for the model parameters of interest. The model parameters were estimated using a Markov chain Monte Carlo method as implemented in the WinBUGS software package [19].

## Results

### Study selection

**Fig. 1:**
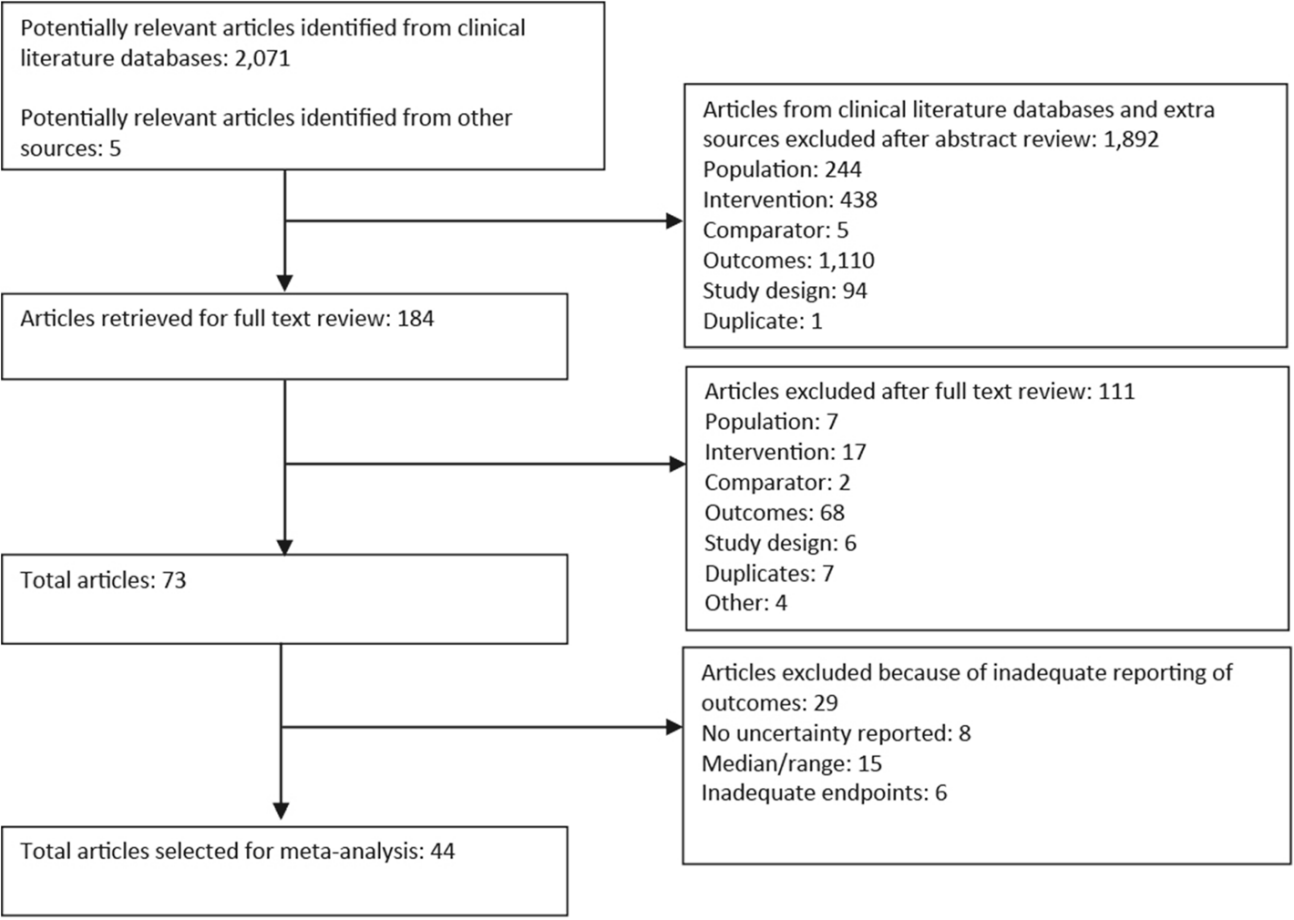
Flow diagram of study selection

The study selection process, including the reasons for exclusion, is summarized in Fig. 1. The literature search identified 2076 potentially relevant studies, although the first review excluded 1892 (91 %) of these. The full-text review of the 184 remaining studies excluded another 111 studies. Of the 73 articles meeting the selection criteria, another 29 studies were excluded because of insufficient data on the outcomes of interest during follow-up. Overall, 44 studies were included [20–63].

### Study characteristics

Information on key study and patient characteristics are presented in Tables 1 and 2. All 44 studies were RCTs, except for one retrospective [31] and two prospective cohort studies [33, 52], and were published between 1982 and 2009, with follow-up periods ranging from 24 weeks to 2 years. Twelve studies concerned the DMARD-IR population [20–31] and the remaining 32 studies concerned the non-DMARD-IR population [32–63]. Only two studies provided data on the Larsen score for the DMARD-IR population, while 10 provided data on the (modified) TSS in this population. For the non-DMARD-IR population, 17 studies provided data on Larsen score only, 14 studies provided data on the (modified) TSS, and one study provided data on both. As some of the studies contributed data from more than one arm (three studies in the DMARD-IR population [21, 22, 31] and 13 studies in the non-DMARD-IR population [35, 36, 38, 41, 48, 50, 51, 54, 57, 59, 61–63]), the total number of treatment arms included in the analyses was 63: 16 for DMARD-IR and 47 for non-DMARD-IR. Among the 47 arms that formed the non-DMARD-IR population, only 12 included patients who had been previously exposed to DMARDs. Hence, the majority of patients in this population could be considered DMARD-naïve.

**Table 1:** Key study characteristics

Study [Ref]	Study design	Follow-up period	Reassignment in studies of biologics	Larsen	JSN	ES	TSS	Outcomes of interest
DMARD-IR population								
Breedveld et al. 2004 [20]	RCT	NA	No reassignment; MTX dose increase for non-responders	No	No	No	Yes	Modified TSS
Hamdy et al. 1987 [21]	Double-blind RCT	1.5 years	NA	No	No	No	Yes	TSS
Jeurissen et al. 1991 [22]	Double-blind RCT	48 weeks	NA	No	No	No	Yes	Modified TSS
Keystone et al. 2004 [23]	Double-blind RCT	1 year	No reassignment; MTX dose increase for non-responders	No	Yes	Yes	Yes	Modified TSS
Keystone et al. 2008 [24]	Double-blind RCT	1 year	No reassignment	No	Yes	Yes	Yes	Modified TSS
Lipsky et al. 2000 [25]	RCT	1 year	No reassignment	No	Yes	Yes	Yes	Modified TSS
Maini et al. 2004 [26]	Double-blind RCT	2 years	No reassignment	No	Yes	Yes	Yes	Modified TSS
Sany et al. 1990 [27]	RCT	2 years	NA	Yes	No	No	No	Larsen scores
Smolen et al. 2009 [28]	RCT	2 years	No reassignment	No	Yes	Yes	Yes	Modified TSS
van der Heijde et al. 2006 [29]	Double-blind RCT	2 years	No reassignment	No	Yes	Yes	Yes	Modified TSS
van Holten et al. 2005 [30]	Double-blind RCT	24 weeks	NA	No	No	No	Yes	Modified TSS
Wick et al. 2005 [31]	Retrospective cohort	NA	NA	Yes	No	No	No	Larsen scores
Non-DMARD-IR population								
Bathon et al. 2000 [32]	RCT	1 year	No reassignment	No	Yes	Yes	Yes	Modified TSS
Boiardi et al. 1999 [33]	Prospective cohort	1 year	NA	Yes	No	No	No	Larsen scores
Breedveld et al. 2006 [34]	Double-blind RCT	2 years	No reassignment; MTX dose increase for non-responders	No	Yes	Yes	Yes	Modified TSS
Choy et al. 2008 [35]	Double-blind RCT	2 years	NA	Yes	No	No	No	Larsen scores
Cohen et al. 2001 [36]	Double-blind RCT	2 years	NA	No	Yes	Yes	Yes	Modified TSS
Cohen et al. 2008 [37]	Double-blind RCT	1 year	NA	No	Yes	Yes	No	Modified TSS
Emery et al. 2000 [38]	Double-blind RCT	2 years	NA	Yes	No	No	No	Larsen scores
Emery et al. 2008 [39]	Double-blind RCT	1 year	No reassignment	No	No	No	Yes	Modified TSS
Emery et al. 2009 [40]	Double-blind RCT	2 years	No reassignment	No	Yes	Yes	Yes	Modified TSS
Ferraccioli et al. 1996 [41]	Open-label RCT	1 year	NA	Yes	No	No	No	Larsen scores
Ferraccioli et al. 1997 [42]	Open-label RCT	1 year	NA	Yes	No	No	No	Larsen scores
Goldbach-Mansky et al. 2009 [43]	RCT	24 weeks	NA	No	Yes	Yes	Yes	Modified TSS
Hannonen et al. 1993 [44]	Double-blind RCT	48 weeks	NA	No	Yes	Yes	Yes	Modified TSS
Hetland et al. 2006 [45]	Double-blind RCT	1 year	NA	Yes	No	No	No	Larsen scores
Hetland et al. 2008 [46]	Double-blind RCT	2 years	NA	No	Yes	Yes	Yes	Modified TSS
Landewé et al. 2002 [47]	Double-blind RCT	1 year	NA	No	No	No	Yes	Modified TSS
Larsen et al. 2001 [48]	Double-blind RCT	24 weeks	NA	Yes	No	No	No	Larsen scores
Maravic et al. 1999 [49]	Open-label RCT	1 year	NA	Yes	No	No	Yes	Larsen scores and TSS
Marchesoni et al. 2002 [50]	First phase was open and uncontrolled; second phase was single-blind RCT	2 years	NA	Yes	Yes	Yes	Yes	Larsen scores and TSS
Peltomaa et al. 1995 [51]	RCT	1 year	NA	Yes	No	No	No	Larsen scores
Sanmarti et al. 2003 [52]	Prospective cohort	1 year	NA	Yes	No	No	No	Larsen scores
Sarzi-Puttini et al. 2005 [53]	RCT	1 year	NA	Yes	No	No	No	Larsen scores
Scott et al. 1985 [54]	RCT	1 year	NA	Yes	No	No	No	Larsen scores
Scott et al. 1989 [55]	RCT	1 year	NA	Yes	No	No	No	Larsen scores
Scott et al. 1990 [56]	RCT	2 years	NA	Yes	No	No	No	Larsen scores
Smith et al. 1982 [57]	Open-label RCT	2 years	NA	Yes	No	No	No	Larsen scores
St Clair et al. 2004 [58]	RCT	2 years	No reassignment	No	Yes	Yes	Yes	Modified TSS,
Strand et al. 1999 [59]	Double-blind RCT	1 year	NA	No	Yes	Yes	Yes	Modified TSS
Svensson et al. 2003 [60]	Double-blind RCT	1.5 years	NA	Yes	No	No	No	Larsen scores
Svensson et al. 2005 [61]	Open-label RCT	2 years	NA	No	Yes	Yes	Yes	Modified TSS
van Riel et al. 1986 [62]	Single-blind RCT	1 year	NA	Yes	No	No	No	Modified Larsen scores
Wassenberg et al. 2005 [63]	Double-blind RCT	2 years	NA	No	Yes	Yes	Yes	Modified TSS

*DMARD* disease-modifying anti-rheumatic drug, *DMARD*-*IR* patient population with moderate-to-severe RA with a history of inadequate response to non-biologic DMARDs who are currently treated with one (other) non-biologic DMARD, *ES* Erosion Subscore of TSS, *JSN* Joint Space Narrowing Subscore of TSS, *MTX* methotrexate, *NA* not available, *non*-*DMARD*-*IR* patient population with moderate to severe RA without a history of inadequate response to a DMARD who are currently receiving palliative care (non-steroidal anti-inflammatory drugs, analgesics, low-dose glucocorticoids) or are being minimally treated with one non-biologic DMARD, *RA* rheumatoid arthritis, *Ref* reference, *RCT* randomized controlled trial, *TSS* modified Total Sharp Score

**Table 2:** Patient characteristics at baseline

Study [Ref] Patient type	Treatment (No. patients, ITT)	Female, %	Mean age (SD), years	RF+, %	Mean disease duration (SD), months	Mean CRP (SD), mg/dL	Mean ESR (SD), mm/h	Mean score (SD)							
								HAQ (0–3)	DAS-28	SJC	TJC	TSS	JSN	ES	Larsen
DMARD-IR population															
Breedveld et al. 2004 [20] DMARD-experienced	MTX (*n* = 88)	78	54^a^	NA	100.8	2.6^a^	42^a^	1.8^a^	NA	20^a^	31^a^	52^a^	28^a^	24^a^	NA
Hamdy et al. 1987 [21] DMARD-experienced	AZA (*n* = 21)	NA	NA	NA	NA	NA	44.8	NA	NA	21.6	NA	53.1	NA	NA	NA
	MTX (*n* = 21)	NA	NA	NA	NA	NA	41.5	NA	NA	22.2	NA	44.5	NA	NA	NA
Jeurissen et al. 1991 [22] DMARD-experienced	AZA (*n* = 33)	52	56 (9)	NA	112.8 (67.2)	NA	60.2 (27.9)	NA	NA	18.7 (7.0)	23.0 (10.7)	60.5	NA	NA	NA
	MTX (*n* = 31)	84	57 (10)	NA	153.6 (120.0)	NA	57.5 (27.9)	NA	NA	18.8 (7.2)	22.5 (10.1)	62.7	NA	NA	NA
Keystone et al. 2004 [23] DMARD-experienced	MTX (*n* = 200)	73	56 (12)	89.5	130.8 (105.6)	1.8 (2.1)	NA	1.5 (0.6)	NA	19 (9.5)	28.1 (13.8)	66.4 (47.4)	29.2 (24.5)	37.2 (25.8)	NA
Keystone et al. 2008 [24] MTX-experienced	MTX (*n* = 199)	84	52 (11)	82.8	74.4 (52.8)	1.6^a^	NA	1.7 (0.6)	7.0^a^	21.2 (9.7)	29.8 (13.0)	NA	NA	NA	NA
Lipsky et al. 2000 [25] DMARD-IR	MTX (*n* = 88)	80	51 (12)	77.0	132.0 (96.0)	4.0 (4.2)	49.0 (25.0)	1.7 (0.6)	NA	21 (12.0)	31.0 (18.0)	82.0 (77.0)	NA	NA	NA
Maini et al. 2004 [26] DMARD-experienced	MTX (*n* = 88)	78	54	NA	NA	2.6	NA	1.8	NA	20.0	31.0	NA	28.3	23.5	NA
Sany et al. 1990 [27] DMARD-experienced	MTX (*n* = 41)	88	54 (27–78)^b^	82.9	154.8	NA	62.8 (34.3)	NA	NA	9.1 (2.8)	NA	NA	NA	NA	83.8 (24.4)
Smolen et al. 2009 [28] MTX-experienced	MTX (*n* = 127)	84	52 (12)	78.2	67.2 (46.8)	1.4	40.8	1.6 (0.6)	6.8 (0.9)	21.9 (9.7)	30.4 (13.4)	46.5 (58.6)	23.4 (27.7)	23.1 (32.1)	NA
van der Heijde et al. 2006 [29] MTX-experienced	MTX (*n* = 228)	76	52	NA	NA	2.6	NA	1.7	NA	22.7	33.2	35.5	NA	NA	NA
van Holten et al. 2005 [30] MTX-experienced	MTX (*n* = 73)	66	54 (12)	71.2	49.2 (28.8)	NA	NA	NA	NA	NA	NA	NA	NA	NA	NA
Wick et al. 2005 [31] DMARD-experienced	MTX (*n* = 56)	71	NA	NA	6.8 (3.9)	3.2 (4.0)	29.5 (20.3)	1.1 (0.6)	5.4 (1.1)	11 (5.6)	9.8 (6.2)	NA	NA	NA	15.6 (9.6)
	SSZ (*n* = 55)	56	NA	NA	6.5 (3.6)	1.9 (18.0)	28.2 (23.1)	0.8 (0.5)	4.8 (1.1)	10.3 (5.8)	7.5 (6.0)	NA	NA	NA	12.9 (9.4)
	Gold (*n* = 19)	63	NA	NA	7.0 (4.4)	1.1 (9.0)	16.3 (9.6)	0.8 (0.5)	4.1 (0.9)	4.5 (3.5)	4.6 (3.7)	NA	NA	NA	9.6 (7.0)
Non-DMARD-IR population															
Bathon et al. 2000 [32] DMARD-experienced	MTX (*n* = 217)	75	49 (13)	89.0	12.0 (11.0)	3.7 (4.4)	NA	NA	NA	24 (11.9)	30.0 (16.1)	12.9 (13.8)	5.4 (6.1)	7.5 (9.2)	NA
Boiardi et al. 1999 [33] DMARD-experienced	MTX (*n* = 20)	90	64 (13)	50.0	69.0 (90.0)	2.5 (2.1)	39.0 (22.0)	NA	NA	8.9 (3.8)	NA	NA	NA	NA	39.4 (34.6)
Breedveld et al. 2006 [34] DMARD-naïve	MTX (*n* = 257)	74	52 (13)	NA	9.6 (10.8)	4.0 (4.0)	NA	1.5 (0.6)	6.3 (0.9)	22.1 (11.7)	32.3 (14.3)	21.9 (22.2)	8.2 (10.7)	13.6 (13.6)	NA
Choy et al. 2008 [35] Early RA; DMARD-experienced	MTX (*n* = 117)	67	54 (21–89)^b^	66.0	2.7 (3.8)	NA	NA	1.5 (0.7)	5.8 (1.2)	NA	NA	NA	NA	NA	7^a^ (3–15)^b^
	MTX (*n* = 115)	78	54 (27–84)^b^	66.0	5.1 (5.8)	NA	NA	1.6 (0.7)	5.8 (1.4)	NA	NA	NA	NA	NA	6^a^ (2–20)^b^
Cohen et al. 2001 [36] MTX-naïve	LEF (*n* = 190)	73	54	NA	82.8	2.2 (2.8)	38.3 (26.0)	0.7 (0.5)	NA	13.3 (6.3)	13.4 (5.6)	23.8 (38.5)	13.5 (17.2)	10.3 (25.6)	NA
	MTX (*n* = 190)	74	53	NA	78.0	2.0 (1.9)	35.9 (25.7)	0.7 (0.5)	NA	13 (5.4)	14.3 (6.5)	25.1 (42.3)	14.5 (21.7)	10.6 (22.9)	NA
Cohen et al. 2008 [37] MTX-experienced	DMARD (*n* = 75)	83	57 (11)	78.0	116.4 (97.2)	NA	NA	NA	NA	NA	NA	29.9 (34.7)	13.3 (18.9)	16.6 (17.2)	NA
Emery et al. 2000 [38] DMARD-experienced	LEF (*n* = 501)	71	58 (10)	NA	44.4 (38.4)	NA	NA	NA	NA	15.8 (6.0)	17.2 (6.8)	NA	NA	NA	1.3 (0.5)
	MTX (*n* = 498)	71	58 (11)	NA	45.6 (42.0)	NA	NA	NA	NA	16.5 (5.9)	17.7 (6.7)	NA	NA	NA	1.3 (0.5)
Emery et al. 2008 [39] MTX-naïve	MTX (*n* = 263)	73	52 (1)	NA	9.3 (0.4)	3.7 (3.4)	49.3 (24.1)	1.6 (0.7)	6.5 (1.0)	17.6 (10.0)	24.8 (14.5)	NA	NA	NA	NA
Emery et al. 2009 [40] MTX-naïve	MTX (*n* = 257)	74	52 (13)	84.0	10.1 (10.7)	4.0 (4.0)	NA	1.5 (0.7)	6.3 (0.9)	22.1 (11.7)	32.3 (14.3)	21.9 (22.2)	8.2 (10.7)	13.6 (13.5)	NA
Ferraccioli et al. 1996 [41] DMARD-naïve	CSA (*n* = 141)	77	48 (12)	NA	16.8 (14.4)	8.3 (16.7)	46.9 (25.2)	NA	NA	14.9 (7.5)	23.8 (9.6)	NA	NA	NA	25.0 (23.0)
	Anti-malarial/Gold/D-penicill-amine/SSZ (*n* = 143)	78	51 (12)	NA	15.6 (13.2)	8.5 (17.5)	47.5 (30.8)	NA	NA	14.7 (7.8)	23.2 (9.8)	NA	NA	NA	23.8 (24.1)
Ferraccioli et al. 1997 [42] DMARD-naïve	CSA (*n* = 167)	78	48 (12)	NA	16.8 (14.4)	7.7 (15.5)	48.5 (26.2)	NA	NA	15.2 (7.7)	23.8 (9.4)	NA	NA	NA	26.0 (21.8)
	Anti-malarial/Gold/D-penicill-amine/SSZ (*n* = 173)	79	50 (12)	NA	15.6 (14.4)	8.7 (18.0)	47.6 (30.7)	NA	NA	15.1 (7.7)	23.9 (9.7)	NA	NA	NA	25.0 (22.2)
Goldbach-Mansky et al. 2009 [43] MTX-experienced	SSZ (*n* = 61)	87	52 (12)	NA	NA	2.5 (2.9)	51.0 (23.0)	NA	7.0 (1.0)	22 (13.0)	33.0 (17.0)	21.4 (31.2)	13.3	8.1 (0.4)	NA
Hannonen et al. 1993 [44] DMARD-naïve	SSZ (*n* = 38)	61	52 (22–78)^b^	65.8	4.7	2.7 (3.0)	37.7 (21.3)	NA	NA	6.8 (3.3)	NA	1.9 (0–14.0)^b^	1.1 (0–8.0)^b^	0.8 (0–6.0)^b^	NA
Hetland et al. 2006 [45] Early RA; DMARD-naïve	MTX (*n* = 80)	70	51^a^ (39.5-62.5)^c^	59.0	46.8	1.9^a^	27^a^	0.9^a^	5.5 (1.3)	11^a^ (6–15)^c^	14^a^ (8–20)^c^	NA	NA	NA	4.6 (7.4)
Hetland et al. 2008 [46] Early RA; MTX-experienced	MTX (*n* = 80)	NA	NA	NA	NA	1.9^a^	27^a^	0.9^a^	5.7^a^	11^a^ (6–15)^c^	14^a^ (8–20)^c^	4.8 (5.9)	1.7 (3.8)	3.1 (4.1)	NA
Landewé et al. 2002 [47] DMARD-naïve	SSZ (*n* = 74)	53	NA	71.0	3.6	NA	NA	1.4 (0.7)	6.1 (1.1)	NA	NA	5^a^ (1–10)^c^	NA	NA	NA
Larsen et al. 2001 [48] DMARD-experienced	LEF (*n* = 133)	76	58 (11)	NA	96.0 (108.0)	NA	NA	NA	NA	NA	NA	NA	NA	NA	1.5 (0.7)
	SSZ (*n* = 133)	69	59 (11)	NA	84.0 (120.0)	NA	NA	NA	NA	NA	NA	NA	NA	NA	1.4 (0.7)
Maravic et al. 1999 [49] MTX-naïve	MTX (*n* = 29)	72	49 (15)	37.9	6.6	1.9^a^	36^a^	NA	NA	12^a^ (3–27)^b^	NA	4.4	NA	NA	15.8 (15.9)
Marchesoni et al. 2002 [50] DMARD-naïve	CSA (*n* = 22)	NA	50 (12)	NA	9.6 (6.0)	0.8 (1.1)	29.2 (17.4)	0.5 (0.5)	3.2 (1.2)	3.2 (4.0)	4.3 (5.2)	NA	NA	NA	5.1 (7.0)
	MTX (*n* = 27)	NA	50 (12)	NA	9.6 (6.0)	0.6 (1.3)	29.4 (13.2)	0.4 (0.5)	3.5 (1.0)	3.1 (4.6)	4.8 (5.7)	NA	NA	NA	5.0 (8.8)
Peltomaa et al. 1995 [51] DMARD-naïve	Gold (*n* = 70)	77	45	NA	7.7	2.7	35.0	NA	NA	NA	NA	NA	NA	NA	5.5 (0–22)^b^
	SSZ (*n* = 58)	71	49	NA	5.8	2.8	41.0	NA	NA	NA	NA	NA	NA	NA	8.9 (0–34)^b^
Sanmarti et al. 2003 [52] DMARD-naïve	Gold/MTX (*n* = 60)	78	52 (16)	78.3	9.5 (6.5)	2.9 (3.1)	45.3 (27.7)	1.0 (0.5)	5.8 (0.8)	8.0 (4.1)	10.2 (5.5)	NA	NA	NA	1.9 (3.3)
Sarzi-Puttini et al. 2005 [53] DMARD-naïve	CSA (*n* = 36)	78	51 (10)	66.7	14.8 (8.4)	3.0 (1.8)	45.0 (20.0)	1.3 (0.6)	NA	12.2 (5.6)	15.31 (6.41)	NA	NA	NA	14.5 (10.0)
Scott et al. 1985 [54] DMARD-naïve	DMARD (*n* = 56)	55	56	NA	NA	4.9	56.5	NA	NA	NA	NA	NA	NA	NA	52.0 (27.7)
	NSAID (*n* = 15)	60	52	NA	NA	1.9	22.6	NA	NA	NA	NA	NA	NA	NA	41.3 (34.5)
Scott et al. 1989 [55] DMARD-naïve	Gold (*n* = 49)	61	55 (3)	NA	24.0	0.5 (1.1)	57.0 (7.0)	NA	NA	NA	NA	NA	NA	NA	33.9 (9.8)
Scott et al. 1990 [56] DMARD-naïve	D-penicill-amine (*n* = 20)	NA	NA	NA	NA	6.0	NA	NA	NA	NA	NA	NA	NA	NA	25.6
	HCQ (*n* = 23)	NA	NA	NA	NA	6.2	NA	NA	NA	NA	NA	NA	NA	NA	25.5
Smith et al. 1982 [57] DMARD-naïve	Gold (*n* = 26)	69	45	80.8	90.0	NA	89.8	NA	NA	NA	NA	NA	NA	NA	54.2 (6.0)
	Gold (*n* = 26)	62	46	65.4	76.8	NA	83.4	NA	NA	NA	NA	NA	NA	NA	47.4 (5.0)
St Clair et al. 2004 [58] DMARD-experienced	MTX (*n* = 282)	75	50 (13)	71.0	10.8 (8.4)	2.6 (2.9)	43.0 (28.0)	1.5 (0.6)	6.7 (1.0)	22.0 (11.0)	34 (15.0)	11.3 (15.9)	3.0 (4.8)	8.3 (12.3)	NA
Strand et al. 1999 [59] MTX-naïve	LEF (*n* = 182)	73	54 (12)	64.8	84.0 (103.2)	2.1 (2.5)	38.4 (26.8)	0.8 (0.6)	NA	13.7 (6.0)	15.5 (6.4)	23.1 (34.0)	14.2 (18.9)	9.0 (19.6)	NA
	MTX (*n* = 182)	75	53 (12)	59.4	78.0	1.9 (1.9)	33.8 (25.4)	0.8 (0.5)	NA	13.0 (5.7)	15.8 (6.9)	22.8 (39.0)	14.7 (23.3)	8.1 (18.4)	NA
Svensson et al. 2003 [60] Early RA; DMARD-experienced	MTX (*n* = 50)	58	52	62.0	9.0	5.4	49.9	1.2	NA	19.8	NA	NA	NA	NA	7.5 (10.8)
Svensson et al. 2005 [61] DMARD-naïve	DMARD (*n* = 119)	65	51 (14)	66.0	6.5 (3.5)	2.2	NA	1.0 (0.6)	5.3 (1.1)	NA	NA	4.1 (9.2)	2.2 (4.6)	1.9 (5)	NA
	DMARD (*n* = 131)	63	59 (13)	66.0	5.8 (2.9)	2.2	NA	1.0 (0.7)	5.4 (1.0)	NA	NA	4.8 (9.6)	2.9 (6.4)	1.9 (4)	NA
Van Riel et al. 1986 [62] DMARD-naïve	Gold (*n* = 22)	77	50 (12)	NA	37.2 (38.4)	NA	56.0 (36.0)	NA	NA	NA	NA	NA	NA	NA	30.9 (12.5)
	Gold (*n* = 18)	72	55 (8)	NA	51.6 (52.8)	NA	53.0 (37.0)	NA	NA	NA	NA	NA	NA	NA	27.6 (12.5)
Wassenberg et al. 2005 [63] MTX-naïve	Gold (66 %)/MTX (34 %) (*n* = 80)	25	53 (13)	43.0	8.6 (6.7)	NA	44.5 (24.9)	NA	NA	NA	NA	11.9 (2.1)	6.0 (1.3)	6.0 (1.08)	NA
	Gold (59 %)/MTX (41 %) (*n* = 86)	35	50 (13)	47.0	9.3 (6.6)	NA	40.1 (24.6)	NA	NA	NA	NA	9.4 (1.9)	4.5 (1.2)	4.7 (0.87)	NA

*AZA* azathioprine, *CRP* C-reactive protein, *CSA* ciclosporin A, *DAS*-*28* disease activity score in 28 joints, *DMARD* disease-modifying anti-rheumatic drug, *DMARD*-*IR* patient population with moderate-to-severe RA with a history of inadequate response to non-biologic DMARDs who are currently treated with one (other) non-biologic DMARD, *ES* Erosion Subscore of TSS, *ESR* erythrocyte sedimentation rate, *gold* gold salts, *HAQ* Health Assessment Questionnaire, *HCQ* hydroxychloroquine, *ITT* intent to treat, *JSN* Joint Space Narrowing Subscore of TSS, *LEF* leflunomide, *MTX* methotrexate, *NA* not available, *non*-*DMARD*-*IR* patient population with moderate-to-severe RA without a history of inadequate response to a DMARD who are currently receiving palliative care (NSAIDs, analgesics, low-dose glucocorticoids) or are being minimally treated with one non-biologic DMARD, *NSAID* non-steroidal anti-inflammatory drug, *RA* rheumatoid arthritis, *Ref* reference, *RF*+ rheumatoid factor positive, *SJC* swollen joint count, *SSZ* sulfasalazine, *TJC* tender joint count, *TSS* modified Total Sharp Score

^a^Median. ^b^Range. ^c^Interquartile range

The patients received the following treatments g : MTX (in 12 treatment arms across the studies); AZA (2); SSZ (1); and gold salts (1) for the DMARD-IR population; and MTX (16); gold salts (9); SSZ (5); LEF (four 4); CSA (4); HCQ (1); D-penicillamine (1); antimalarials, D-penicillamine, SSZ, or gold salts (2); any one of a list of non-biologic DMARDs (4); and NSAIDs (4) for the non-DMARD-IR population. The number of patients included ranged from 29 to 228 for DMARD-IR arms and from 20 to 501 for non-DMARD-IR arms.

### Patient characteristics

The DMARD-IR and the non-DMARD-IR populations showed comparable distributions for gender, age, baseline C-reactive protein (CRP) level, and erythrocyte sedimentation rate (ESR). On average, 73 % of the patients were women in the DMARD-IR studies versus 70 % in the non-DMARD-IR studies; the average ages were 54 and 52 years, respectively. The median of the reported baseline CRP level was 2.2 mg/dL across the DMARD-IR studies and 2.6 mg/dL across the non-DMARD-IR studies. The median of the reported baseline ESR was 43 mm/h across DMARD-IR studies and 44 mm/h across non-DMARD-IR studies. There was a large variation in baseline CRP level and ESR across the non-DMARD-IR studies. As expected, the disease duration was skewed and longer among the DMARD-IR patients (median of 88 months) versus the non-DMARD-IR patients (median of 15 months). For the DMARD-IR population, the median of the reported baseline Health Assessment Questionnaire (HAQ) score across the studies was 1.7; the HAQ scale ranges from zero (no disability) to three (completely disabled). The median baseline TSS for the DMARD-IR population was 53. For the non-DMARD-IR population the median of the reported HAQ scores across studies was 1.0 and the median TSS was 11.9.

### Joint structural deterioration over time in the DMARD-IR population

**Fig. 2:**
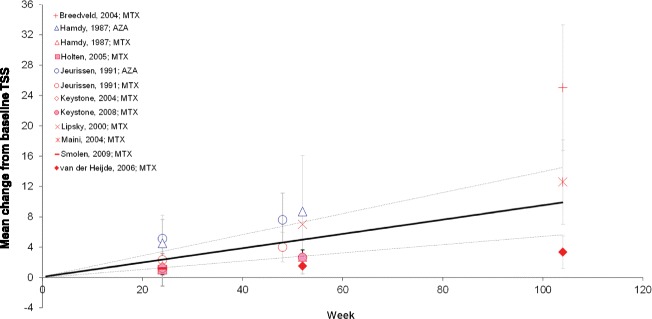
Mean change from baseline in TSS in the DMARD-IR population. Data are as observed in individual studies and estimated with meta-analysis. Solid line represents the mean estimate for a given treatment arm and the dashed lines show the corresponding 95 % credible interval. AZA: azathioprine; DMARD-IR: patient population with moderate-to-severe rheumatoid arthritis with a history of inadequate response to disease-modifying anti-rheumatic drugs (DMARDs) who are currently treated with one (other) non-biologic DMARD; MTX: methotrexate; TSS: modified Total Sharp Score

The mean CFB in TSS within the DMARD-IR population, as obtained from the individual studies, is presented in Fig. 2. These results were combined with a random-effects meta-analysis (Additional file 2, Model 1), where the change in TSS over time developed in a linear fashion from 1.14 at Week 12 to 9.84 at Week 104 (Table 3). There was a high probability that continuation of treatment with any one non-biologic DMARD in the setting of inadequate response would result in deterioration of the joint structure over time.

**Table 3:** Mean change from baseline in TSS and subscores in the DMARD-IR population as estimated with meta-analysis

	Week 12			Week 24			Week 52			Week 104		
	CFB	95 % CrI	Probability of deterioration relative to baseline	CFB	95 % CrI	*p*-value	CFB	95 % CrI	Probability of deterioration relative to baseline	CFB	95 % CrI	Probability of deterioration relative to baseline
TSS												
DMARDs	1.14	(0.66, 1.67)	>0.99	2.27	(1.31, 3.34)	>0.99	4.92	(2.84, 7.23)	>0.99	9.84	(5.68, 14.46)	>0.99
MTX^a^	0.97	(0.49, 1.52)	0.99	1.95	(0.98, 3.05)	0.99	4.22	(2.13, 6.6)	0.99	8.44	(4.26, 13.21)	0.99
AZA^a^	2.16	(2.16, 3.04)	>0.99	4.32	(2.59, 6.07)	>0.99	9.35	(5.62, 13.15)	>0.99	18.70	(11.24, 26.31)	>0.99
Erosion Subscore												
DMARDs	0.51	(0.27, 0.83)	0.99	1.02	(0.55, 1.66)	0.99	2.22	(1.19, 3.6)	0.99	4.43	(2.38, 7.21)	0.99
MTX^a^	0.43	(0.25, 0.69)	0.98	0.87	(0.49, 1.39)	0.98	1.88	(1.07, 3.01)	0.98	3.76	(2.14, 6.01)	0.98
AZA^a^	1.31	(1.31, 1.98)	>0.99	2.62	(1.3, 3.96)	>0.99	5.67	(2.81, 8.57)	>0.99	11.35	(5.61, 17.14)	>0.99
Joint Space Narrowing Subscore												
DMARDs	0.36	(0.09, 0.67)	0.99	0.72	(0.18, 1.33)	0.99	1.57	(0.4, 2.89)	0.99	3.14	(0.80, 5.78)	0.90

*AZA* azathioprine, *CrI* credible interval; *DMARD* disease-modifying anti-rheumatic drug; *DMARD*-*IR* patient population with moderate-to-severe rheumatoid arthritis with a history of inadequate response to non-biologic DMARDs who are currently treated with one (other) non-biologic DMARD, *CFB* change from baseline; *MTX* methotrexate, *p*-*value* probability of joint structural deterioration relative to baseline; *TSS* modified Total Sharp Score

^a^Estimated based on comparative data only, using models for relative treatment effects (see Model 3 and 4 in Additional file 2), which allows comparative interpretation of MTX and AZA findings

Table 3 also presents the results of the analysis (Additional file 2, Model 3) that compared joint deterioration as observed with MTX and AZA. Continuation of treatment with AZA was associated with greater joint deterioration than continuation of treatment with MTX in this DMARD-IR population.

**Fig. 3:**
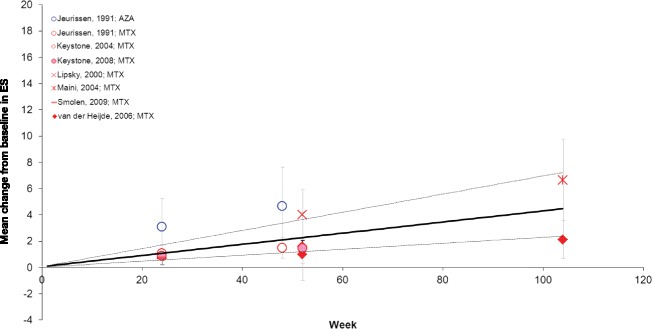
Mean change from baseline in ES in the DMARD-IR population. Data are as observed in individual studies and estimated with meta-analysis. Solid line represents the mean estimate for a given treatment arm and the dashed lines show the corresponding 95 % credible interval. AZA: azathioprine; DMARD-IR: patient population with moderate-to-severe rheumatoid arthritis with a history of inadequate response to disease-modifying anti-rheumatic drugs (DMARDs) who are currently treated with one (other) non-biologic DMARD; ES: Erosion Subscore; MTX: methotrexate

The progression in ES extracted from the individual studies and the pooled results (0.51 at Week 12 and 4.43 at Week 104) obtained with the meta-analysis (Additional file 2, Model 1) are presented in Fig. 3. There was a 98 % to 100 % chance that ES would deteriorate over time when DMARD-IR patients received minimal treatment with a non-biologic DMARD (Table 3). As inferred from the comparative analysis, a greater rate of deterioration was expected with AZA than with MTX.

**Fig. 4:**
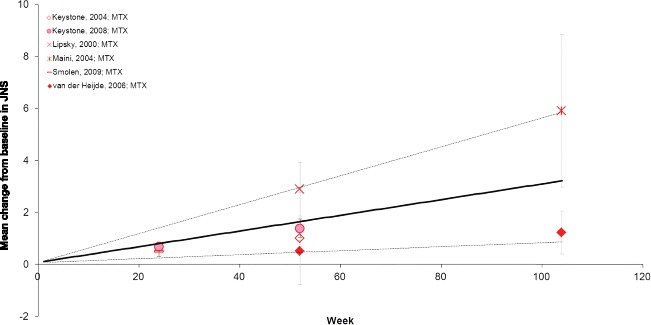
Mean change from baseline in JSN Subscore in the DMARD-IR population. Data are as observed in individual studies and estimated with meta-analysis. Solid line represents the mean estimate for a given treatment arm and the dashed lines show the corresponding 95 % credible interval. DMARD-IR: patient population with moderate-to-severe rheumatoid arthritis with a history of inadequate response to disease-modifying anti-rheumatic drugs (DMARDs) who are currently treated with one (other) non-biologic DMARD; JSN: Joint Space Narrowing; MTX: methotrexate

When DMARD-IR patients were treated with MTX alone mean changes in JSN were 0.36 at Week 12 and 3.14 at Week 104 (Fig. 4; Table 3).

For joint deterioration as measured with the Larsen score, only two studies with sufficient data were available for the DMARD-IR population [27, 31]. As neither study reported repeated intermediate observations, no meta-analysis model for change over time was estimated. At 24 weeks, a deterioration of 0.52 was observed with MTX [27]. In the other study the deterioration varied from 0.53 points with SSZ to 1.06 with gold salts at 52 weeks [31].

### Joint structural deterioration over time in the non-DMARD-IR population

**Fig. 5:**
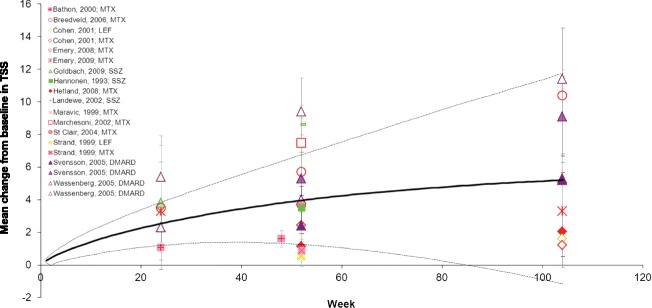
Mean change from baseline in TSS in the non-DMARD-IR population. Data are as observed in individual studies and estimated with meta-analysis. Solid line represents the mean estimate for a given treatment arm and the dashed lines show the corresponding 95 % credible interval. AZA: azathioprine; DMARD: disease-modifying anti-rheumatic drugs; DMARD-IR: patient population with moderate-to-severe rheumatoid arthritis with a history of inadequate response to DMARDs who are currently treated with one (other) non-biologic DMARD; LEF: leflunomide; MTX: methotrexate; SSZ: sulfasalazine; TSS: modified Total Sharp Score

The rate of deterioration in the non-DMARD-IR population was not as great as for the DMARD-IR patients. The progression of the TSS for the non-DMARD-IR population is presented in Fig. 5 and Table 4. Individual study results were combined with a random-effects meta-analysis model, where the change in TSS from baseline developed in a non-linear fashion (fractional polynomial with p1 = p2 = 1. Additional file 2, Model 2) and shows an increase in TSS from 1.56 at 12 weeks to 5.13 at 104 weeks. Up to at least 104 weeks, there was at least a 94 % chance that continuing treatment with one DMARD would result in deterioration of the joint structure in the non-DMARD-IR population (Table 4). An analysis (Additional file 2, Model 4) comparing LEF and MTX based on two head-to-head RCTs indicated a similar rate of deterioration with LEF and MTX [37, 59].

**Table 4:** Mean change from baseline in TSS and subscores plus Larsen Score in the non-DMARD-IR population as estimated with meta-analysis

	Week 12			Week 24			Week 52			Week 104		
	CFB	95 % CrI	Probability of deterioration relative to baseline	CFB	95 % CrI	Probability of deterioration relative to baseline	CFB	95 % CrI	Probability of deterioration relative to baseline	CFB	95 % CrI	Probability of deterioration relative to baseline
TSS												
DMARDs	1.56	(0.79, 2.34)	>0.99	2.49	(1.2, 3.79)	>0.99	3.91	(1.16, 6.7)	>0.99	5.13	(−1.35, 11.67)	0.94
MTX^a^	1.07	(0.62, 1.53)	>0.99	1.76	(1, 2.57)	>0.99	2.89	(1.41, 4.56)	>0.99	4.15	(1.08, 7.63)	>0.99
AZA^a^	0.73	(−0.08, 1.54)	0.96	1.34	(0.17, 2.52)	0.99	2.57	(0.86–4.41)	>0.99	4.55	(1.19, 8.26)	>0.99
Erosion Subscore												
DMARDs	0.69	(0.31, 1.12)	>0.99	1.07	(0.5, 1.67)	>0.99	1.80	(0.76, 2.85)	>0.99	2.93	(0.92, 5.02)	>0.99
MTX^a^	0.64	(0.2, 1.07)	>0.99	0.99	(0.32, 1.68)	>0.99	1.65	(0.35, 3.05)	0.99	2.70	(0.08, 5.53)	0.98
AZA^a^	0.59	(0.13, 1.03)	0.99	0.88	(0.17, 1.59)	0.99	1.41	(0.05, 2.86)	0.98	2.21	(−0.53, 5.14)	0.95
Joint Space Narrowing Subscore												
DMARDs	0.29	(0.17, 0.44)	>0.99	0.59	(0.34, 0.88)	>0.99	1.28	(0.73, 1.9)	>0.99	2.55	(1.45, 3.8)	>0.99
MTX^a^	0.26	(0.05, 0.51)	0.99	0.51	(0.1, 1.02)	0.99	1.11	(0.22, 2.2)	0.99	2.21	(0.45, 4.41)	0.99
AZA^a^	0.24	(0.02, 0.5)	0.98	0.48	(0.05, 1)	0.98	1.04	(0.1, 2.17)	0.98	2.08	(0.21, 4.34)	0.98
(Standardized) Larsen score												
DMARDs	0.08	(0.04–0.11)	>0.99	0.15	(0.08–0.22)	>0.99	0.33	(0.18–0.48)	>0.99	0.65	(0.36–0.96)	>0.99

*AZA* azathioprine, *CFB* change from baseline, *CrI* credible interval, *DMARD* disease-modifying anti-rheumatic drug, *MTX* methotrexate, *non*-*DMARD*-*IR* patient population with moderate-to-severe rheumatoid arthritis without a history of inadequate response to a DMARD who are currently receiving palliative care (non-steroidal anti-inflammatory drugs, analgesics, low-dose glucocorticoids) or are being minimally treated with one non-biologic DMARD, *p*-*value* probability of joint structural deterioration relative to baseline, *TSS* modified Total Sharp Score

^a^Estimated based on comparative data only, using models for relative treatment effects (see Model 3 and 4 in Additional file 2), which allows comparative interpretation of MTX and AZA findings

**Fig. 6:**
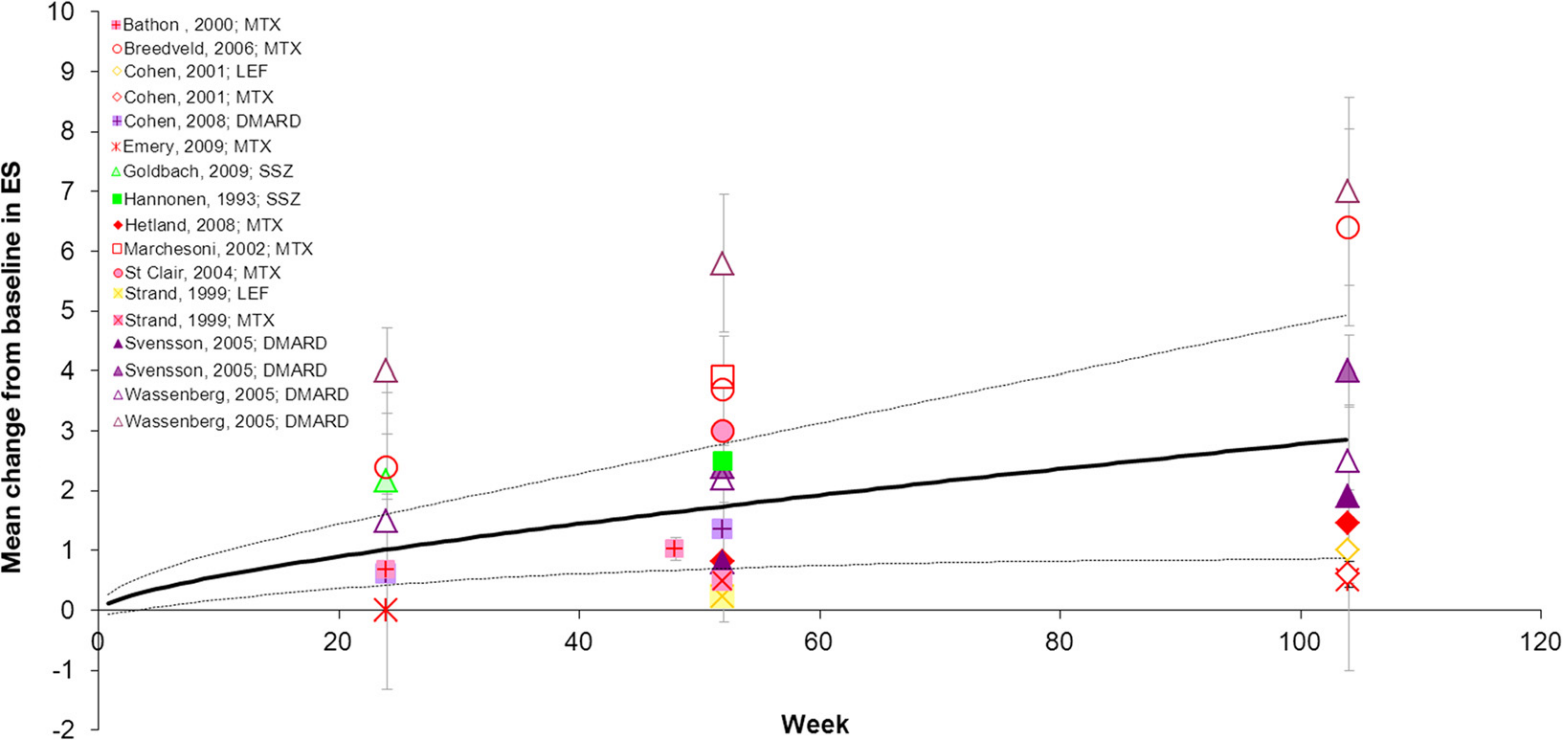
Mean change from baseline in ES in the non-DMARD-IR population. Data are as observed in individual studies and estimated with meta-analysis. Solid line represents the mean estimate for a given treatment arm and the dashed lines show the corresponding 95 % credible interval. DMARD: disease-modifying anti-rheumatic drugs; DMARD-IR: patient population with moderate-to-severe rheumatoid arthritis with a history of inadequate response to DMARDs who are currently treated with one (other) non-biologic DMARD; ES: Erosion Subscore; LEF: leflunomide; MTX: methotrexate; SSZ: sulfasalazine

For ES, the individual study results were also combined with a random-effects non-linear meta-analysis model (fractional polynomial with p1 = 1 and p2 = 0.5; Additional file 2, Model 2) and showed that ES worsened over time from 0.69 at Week 12 to 2.93 at Week 104 when non-DMARD-IR patients continued to receive one traditional DMARD (Fig. 6; Table 4). Comparative analysis showed no difference in rate of deterioration was expected between LEF and MTX (Additional file 2, Model 4).

**Fig. 7:**
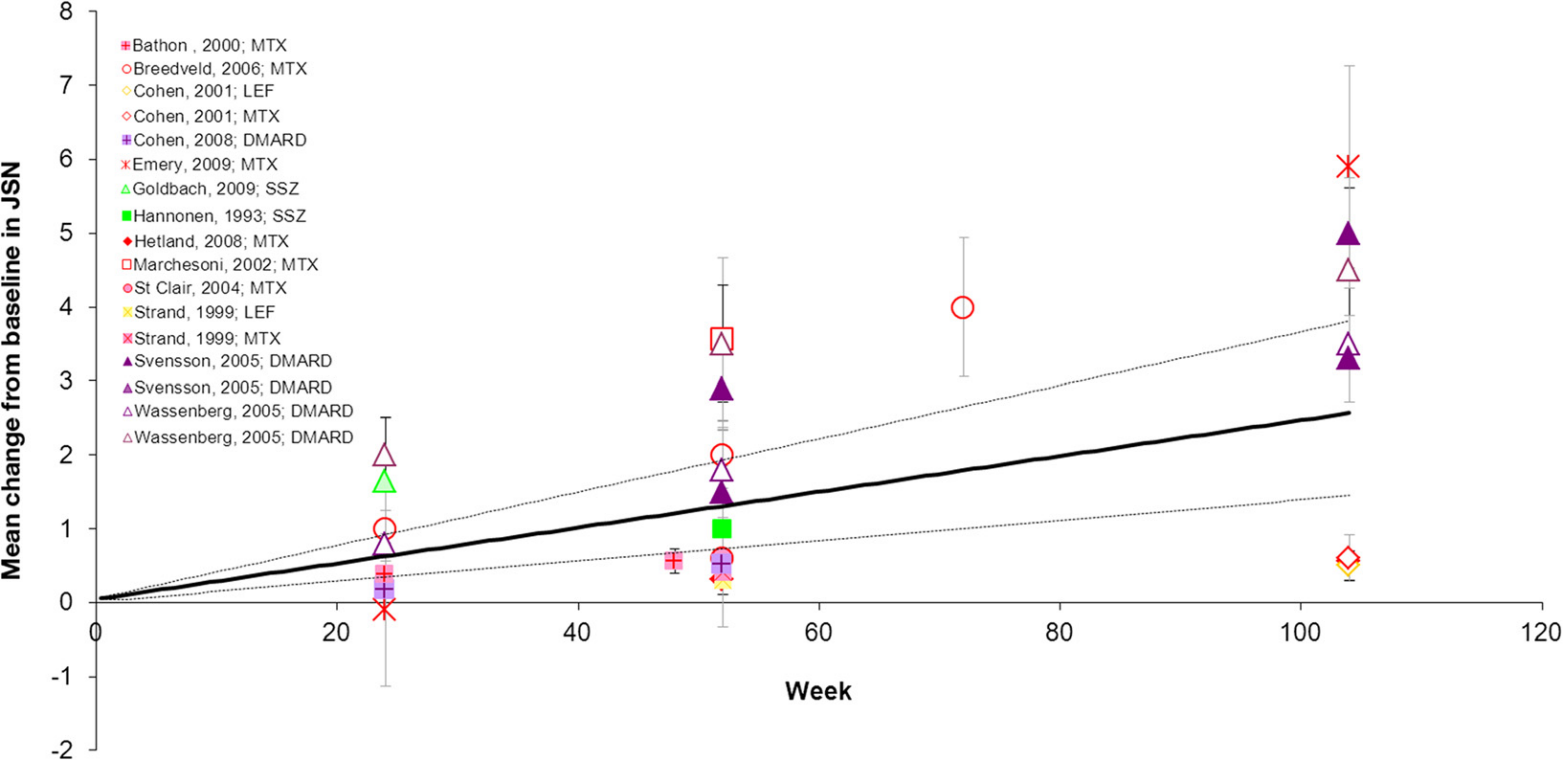
Mean change from baseline in JSN Subscore in the non-DMARD-IR. Data are as observed in individual studies and estimated with meta-analysis. Solid line represents the mean estimate for a given treatment arm and the dashed lines show the corresponding 95 % credible interval. DMARD: disease-modifying anti-rheumatic drugs; DMARD-IR: patient population with moderate-to-severe rheumatoid arthritis with a history of inadequate response to DMARDs who are currently treated with one (other) non-biologic DMARD; JSN: Joint Space Narrowing; LEF: leflunomide; MTX: methotrexate; SSZ: sulfasalazine

For the JSN and the Larsen scores, linear meta-analysis models were appropriate to reflect the deterioration up to 104 weeks (Fig. **7** and Table 4; Additional file 2, Model 1). One study evaluated treatment with NSAIDs only and reported a mean standardized change from baseline in the Larsen score of 0.01 up to 52 weeks [54].

## Discussion

In this study, the development of joint structural deterioration among minimally treated patients with moderate-to-severe RA was estimated based on currently available published data. Estimates were obtained for two populations: a DMARD-IR population that consisted of patients who showed previous inadequate response with non-biologic DMARDs, and a non-DMARD-IR population that consisted of both non-biologic DMARD-naïve and non-biologic DMARD-experienced patients without an inadequate response to any DMARD. In the identified studies, the minimally treated DMARD-IR patients were receiving monotherapy with MTX, AZA, SSZ, or gold salts, with most patients receiving MTX. In the included non-DMARD-IR studies, DMARD treatment consisted of MTX, SSZ, LEF, CSA, HCQ, or D-penicillamine. Only one study was identified in which patients were treated with NSAIDs only, but this study was not included in the meta-analysis. For both populations, treatment with one DMARD resulted in deterioration of joint structure over a 2-year period as measured with the TSS, ES, JSN, and Larsen scores. Under the assumption that the minimal clinically important difference is about 1 % of the maximum of the possible TSS and Larson scores, the estimated changes over a 2 year period in terms of TSS can be considered relevant, in particular for the DMARD-IR population [64, 65]. Depending on the time assessed and the measure examined, the rate of deterioration in the DARD-IR population was about 1.5- to 2-times the rate of deterioration in the non-DMARD-IR population. Based on RCT evidence, the rate of deterioration with AZA was greater than with MTX in the DMARD-IR population. For the non-DMARD-IR population, LEF and MTX showed a similar progression over time.

The greater rate of deterioration observed in the DMARD-IR population compared with the non-DMARD-IR population makes sense, given the negative impact a history of non-biologic-DMARD failure should have on the effectiveness of continuation with a non-biologic DMARD. Related underlying causes for the difference in progression rates are possibly differences in disease duration, rheumatoid factor status, and disease activity.

However, it is important to note that for a subset of the identified studies it was not clear whether the patients were exclusively DMARD-IR. These studies were assigned to the non-DMARD-IR group to make sure that the DMARD-IR group was as homogenous as possible. As such, it is possible that the defined non-DMARD-IR population partly consisted of patients who might have a history of failed treatment with a DMARD. This possible misclassification might have overestimated the deterioration in this group, and should be kept in mind when comparing the degree of joint deterioration in DMARD-IR versus non-DMARD-IR populations.

The relevant studies were identified by means of a systematic search of the literature and included both RCTs and observational designs. Given the objective of the meta-analysis, only those arms of the comparative studies were selected in which patients were treated with NSAIDs or a single DMARD (with or without additional NSAIDs or corticosteroid use). Although many RCTs were included, often only one treatment arm (e.g., MTX-only arm from biologic trials in DMARD-IR populations) was used. As such, there was no difference in the way evidence obtained from observational studies and RCTs was handled. RCTs in which different single non-biologic DMARDs were compared were included and provided the evidence to allow comparisons between DMARDs. Comparative analyses were only possible for AZA versus MTX and for LEF versus MTX. Although RCTs provided comparative data for some other DMARDs, these were not part of a connected network of RCTs and could not, therefore, be used in the planned analyses.

Many of the MTX treatment arms comprising the DMARD-IR population were obtained from RCTs in which a biologic DMARD was evaluated. In the included studies the patients in these MTX arms were not assigned to biologic treatment within the study time horizon; only the MTX dose could be increased in case of non-response. Hence, the observed structural deterioration is a reflection of the limitations of MTX in this population.

The included studies reported joint deterioration at different time points, with outcomes reported up to 2 years of follow-up. With the meta-analysis models used, all the available time points were analyzed simultaneously to estimate a curve reflecting joint structural deterioration over time. It cannot be assumed that extrapolation of these curves beyond this 2-year period is a valid representation of joint structure deterioration over the longer term. The vast majority of studies used the modified Sharp score to analyze joint erosion and space narrowing. The modified score includes feet in the radiographic assessment, in addition to the scoring of wrists and hands as with the original Sharp score [6–10]. The study by Hamdy [21] used the earlier version of the Sharp score. Despite the differences in total score, we included the study by Hamdy in the analysis of the DMARD-IR population. We do not expect this variation in total score to be a cause of large between-study heterogeneity in development of TSS over time. In fact the observed TSS reported by Hamdy is very consistent with the other studies included in that analysis (Fig. 2).

The included studies were characterized by variability in patient characteristics, especially among the non-DMARD-IR studies. As a result, heterogeneity in joint structural deterioration over time was observed. In order to capture this heterogeneity, random-effects models were used; however, these models do not explain the heterogeneity. In the future, it will be of interest to evaluate whether certain patient characteristics are associated with differences in joint deterioration. However, meta-regression analysis where study level data is used to evaluate the impact of patient characteristics on outcomes or treatment effects can be prone to ecological bias [66, 67]. For such an evaluation it is preferred to have access to patient-level data. In this context it would be interesting to evaluate the independent effect of steroid use, disease duration, rheumatoid factor status, and disease activity on joint deterioration, for example.

There is evidence that the patients treated in biologic trials have changed substantially over the past decade. [68]. This is important to consider when using the findings of this meta-analysis to help interpret results of new placebo-controlled trials of biologics. In this context, a limitation of the current analysis is that any potentially relevant studies published after 2009 were not included.

## Conclusions

Based on the currently available published evidence, it can be concluded that minimal treatment of RA with one non-biologic DMARD results in a high risk of deterioration of joint structures among patients who have shown an inadequate response to non-biologic DMARDs, as well as patients that have not (yet) shown an inadequate response. This finding is of relevance when assessing the relative benefit of arresting joint damage with new biologic agents based on findings of placebo-controlled trials in which patients randomized to placebo are ‘rescued’ with active therapy within 6 months.

## Abbreviations

AZA: azathioprine; CFB: change from baseline; CrI: credible interval; CRP: C-reactive protein; CSA: ciclosporin A; DIC: deviance information criterion; DMARD: disease-modifying anti-rheumatic drugs; DMARD-IR: inadequate responder to DMARD therapy; ES: Erosion Subscore; ESR: erythrocyte sedimentation rate; HAQ: Health Assessment Questionnaire; HCQ: hydroxychloroquine; JSN: Joint Space Narrowing; LEF: leflunomide; MTX: methotrexate; NSAID: non-steroidal anti-inflammatory drug; RA: rheumatoid arthritis; RCT: randomized controlled trial; SD: standard deviation; SSZ: sulfasalazine; TSS: Total Sharp Score

## Additional files

Additional file 1: Search strategy. (DOC 23 kb) Additional file 2: Meta analysis models. (DOCX 83 kb)
