# Supplementary material for: Meta-analysis of long-term joint structural deterioration in minimally treated patients with rheumatoid arthritis
Source: BMC Musculoskelet Disord. 2016 Aug 18;17:348. doi: 10.1186/s12891-016-1195-4 (PMC4991055; doi:10.1186/s12891-016-1195-4)
Supplement: Additional file 1: — Search strategy. (DOC 23 kb) [file 12891_2016_1195_MOESM1_ESM.doc]

Additional file 1

Search strategy

#1 rheumatoid arthritis

#2 non-steroidal anti-inflammatory drugs OR NSAIDs OR analgesics OR aspirin OR Ibuprofen OR Celecoxib OR COX OR Celebrex

#3 corticosteroids OR steroids OR glucocorticoids

#4 disease-modifying anti-rheumatic drugs OR DMARD

#5 azathioprine OR Imuran OR Azasan

#6 hydroxychloroquine OR Plaquenil

#7 leflunomide OR Arava

#8 methotrexate OR Rheumatrex OR Trexall OR Folex

#9 minocycline OR Dynacin OR Minocin OR Myrac OR Solodyn

#10 gold OR Ridaura OR Myochrysine OR Solganol

#11 sulfasalazine OR Azulfidine

#12 (# 2 OR # 3 OR # 4 OR # 5 OR # 6 OR # 7 OR # 8 OR # 9 OR # 10 OR # 11)

#13 randomized controlled trial OR randomized-controlled-trial OR controlled-clinical-trial OR randomized OR clinical trial OR random OR RCT OR random allocation OR double-blind method OR single-blind method OR placebo

#14 cohort OR retrospective OR prospective OR database

#15 (#13 OR #14)

#16 ([#1 AND #12] AND #15)

#17 Limit #16 to Humans, Adults

#18 Years: 1970–present

#19 Languages: English, German, French
