# Supplementary material for: Meta-analysis of long-term joint structural deterioration in minimally treated patients with rheumatoid arthritis
Source: BMC Musculoskelet Disord. 2016 Aug 18;17:348. doi: 10.1186/s12891-016-1195-4 (PMC4991055; doi:10.1186/s12891-016-1195-4)
Supplement: Additional file 2: — Meta analysis models. (DOCX 83 kb) [file 12891_2016_1195_MOESM2_ESM.docx]

Additional file 2

Meta analysis models

Meta-analysis models for outcomes over time for one DMARD

Fractional polynomial models are useful when one suspects that the relation between treatment and outcome may be non-linear but wants to preserve the continuous nature of the relation. Fractional polynomials are an extension of polynomial models for determining the functional form of a continuous predictor [14-16]. With the meta-analysis of repeated measures using fractional polynomials, a (non-)linear curve is fitted through the available data points for each study arm. Simultaneously the arm-specific values for parameters describing these curves are pooled by type of intervention taking into account the structure of the evidence base.

Model 1: Random-effects model with a linear development of joint deterioration over time

$$\begin{matrix} y_{jkt}\sim Normal\left(Y_{jkt},\sigma_{jkt}^{2}\right) \\ Y_{jkt}=\beta_{1jk}t\text{ } \\ \beta_{1jk}\sim Normal\left(\mu_{1},\sigma_{1\mu}^{2}\right) \end{matrix}$$

Where $y_{jkt}$ is the observed change from baseline in outcome *y* in study *j*, treatment *k* at time point *t*. $Y_{jkt}$ is the underlying change from baseline in the outcome in study *j*, treatment *k* at time point *t*. $\sigma_{jkt}^{2}$ is its corresponding variance (standard error squared). $\beta_{1jk}$ is study and treatment specific and reflects the linear change in outcome over time relative to baseline. $\beta_{1jk}$ is drawn from a normal distribution with the pooled estimate $\mu_{1}$ and heterogeneity in slope $\sigma_{1\mu}^{2}$ as arguments, which are of key interest for the analyses of these latter two parameters. Within a Bayesian framework prior distributions have to be defined for $\mu_{1}$ and $\sigma_{1\mu}^{2}$.

*Model 2: Random-effects model with a non-linear development* of joint deterioration *over time and two heterogeneity parameters*

Again $Y_{jkt}$ is the underlying change from baseline in the outcome in study *j*, treatment *k* at time point *t*. Now, the development over time is not described by one parameter, but two parameters $\mu_{1}$ and $\mu_{2}$. The vector $\left(\begin{matrix} \beta_{1jk} \\ \beta_{2jk} \end{matrix}\right)$ is trial and treatment specific and reflects the change in outcome over time relative to baseline. The elements $\left(\begin{matrix} \beta_{1jk} \\ \beta_{2jk} \end{matrix}\right)$ are drawn from a bivariate normal distribution with the pooled estimates $\left(\begin{matrix} \mu_{1} \\ \mu_{2} \end{matrix}\right)$. $\Sigma_{\delta}$is the between-study covariance matrix to reflect heterogeneity. represents the heterogeneity for $\beta_{1}$ and reflects the heterogeneity in $\beta_{2}$. $\rho$ is the correlation between these parameters. Of key interest from the analyses are $\left(\begin{matrix} \mu_{1} \\ \mu_{2} \end{matrix}\right)$ and estimates for the heterogeneity, as these describe the joint deterioration over time. Within a Bayesian framework prior distributions have to be defined for $\left(\begin{matrix} \mu_{1} \\ \mu_{2} \end{matrix}\right)$ and $\Sigma_{\mu}$.

Meta-analysis models for differences in outcomes over time between DMARDs

For the analysis of the development of joint structural deterioration by DMARD, first the difference in slope between a particular DMARD and MTX was estimated based on RCT evidence. Second, these difference estimates were combined with the estimated development with MTX over time (obtained with Model 1 or 2) to calculate the expected joint deterioration for the other DMARDs. In all models the development of structural deterioration with MTX was estimated with a random-effects model, and the difference between MTX and other DMARDs with a fixed-effects model given the limited number of comparative RCTs available. The following models were considered for the analysis:

Model 3: Model for the difference in the linear development between different DMARDs compared in RCTs

Where $y_{jkt}$ is the observed change from baseline in outcome *y* in study *j*, intervention *k* at time point *t*. $Y_{jkt}$is the underlying change from baseline in the outcome in study *j*, intervention *k* at time point *t*. $\sigma_{jkt}^{2}$ is corresponding variance (i.e., standard error squared as reported in the individual studies). There are *k* treatments labeled as A, B, C, and so on. $\beta_{1jk}$ is study- and treatment-specific and reflects the linear change (i.e., slope or gradient) in outcome over time relative to baseline. $\beta_{1jk}$ is described as a function of $\mu_{1jb}$ and $d_{1bk}$. $\mu_{1jb}$ is the outcome in study *j* on “baseline treatment” *b* which will vary across studies. The pooled fixed effect of treatment *k* relative to ‘baseline treatment” *b*, $d_{1bk}$, are identified by expressing them in terms of the reference treatment A: $d_{1bk}=d_{1Ak}-d_{1Ab}$ with $d_{1AA}=0$. When all the comparative studies in the evidence base concern one and the same pair-wise comparison (i.e., only AB studies) $d_{1bk}=d_{1Ak}-d_{1Ab}$ is replaced with $d_{1AB}$. Within a Bayesian framework prior distributions have to be defined for the parameters $\mu_{1jb}$ and $d_{1Ak}$. Based on model parameters $\mu_{1}$from Model 1 and $d_{1Ak}$ from Model 3, the expected outcomes for treatment k=A, B, C, D over time can be calculated.

Model 4: Model for the difference in the non-linear development between different DMARDs compared in RCTs

The development of outcomes is now described by the two parameters $\beta_{1jk}$ and $\beta_{2jk}$. The first parameter, $\beta_{1jk}$, is study- and treatment-specific and reflects the linear slope in outcome over time relative to baseline. $\beta_{1jk}$ is a function of nuisance parameter $\mu_{1jb}$ and fixed effects $d_{1Ak}-d_{1Ab}$. The second parameter $\beta_{2jk}$ describes the change in the slope of the development of the outcome over time as a function of a nuisance parameter $\mu_{2jb}$ and fixed effects $d_{2Ak}-d_{2Ab}$ where $d_{1AA}=d_{2AA}=0$. When all the comparative studies in the evidence base concern one and the same pair-wise comparison (i.e., only AB studies) $d_{1Ak}-d_{1Ab}$ and $d_{2Ak}-d_{2Ab}$ are replaced with $d_{1AB}$ and $d_{2AB}$, respectively.

Based on the model parameters $\mu_{1}$ and $\mu_{2}$ from Model 2 and $d_{1Ak}$ and $d_{2Ak}$ from Model 4 the expected outcomes for treatment k=A, B, C, D over time can be calculated.

When it is assumed that the deviation from a linear development over time is the same for all treatments, then $d_{2Ak}-d_{2Ab}$ = 0 can be removed.
